# Supplementary material for: The interactome of intact mitochondria by cross-linking mass spectrometry provides evidence for coexisting respiratory supercomplexes
Source: Mol Cell Proteomics. 2017 Dec 8;17(2):216–32. doi: 10.1074/mcp.RA117.000470 (PMC5795388; doi:10.1074/mcp.RA117.000470)
Supplement: Supplemental Data [file supp_RA117.000470_133922_0_supp_23978_fzffwf.zip › spectra_annotation/mito_spectra_annotation/19-1-2-1-2-1.pdf]

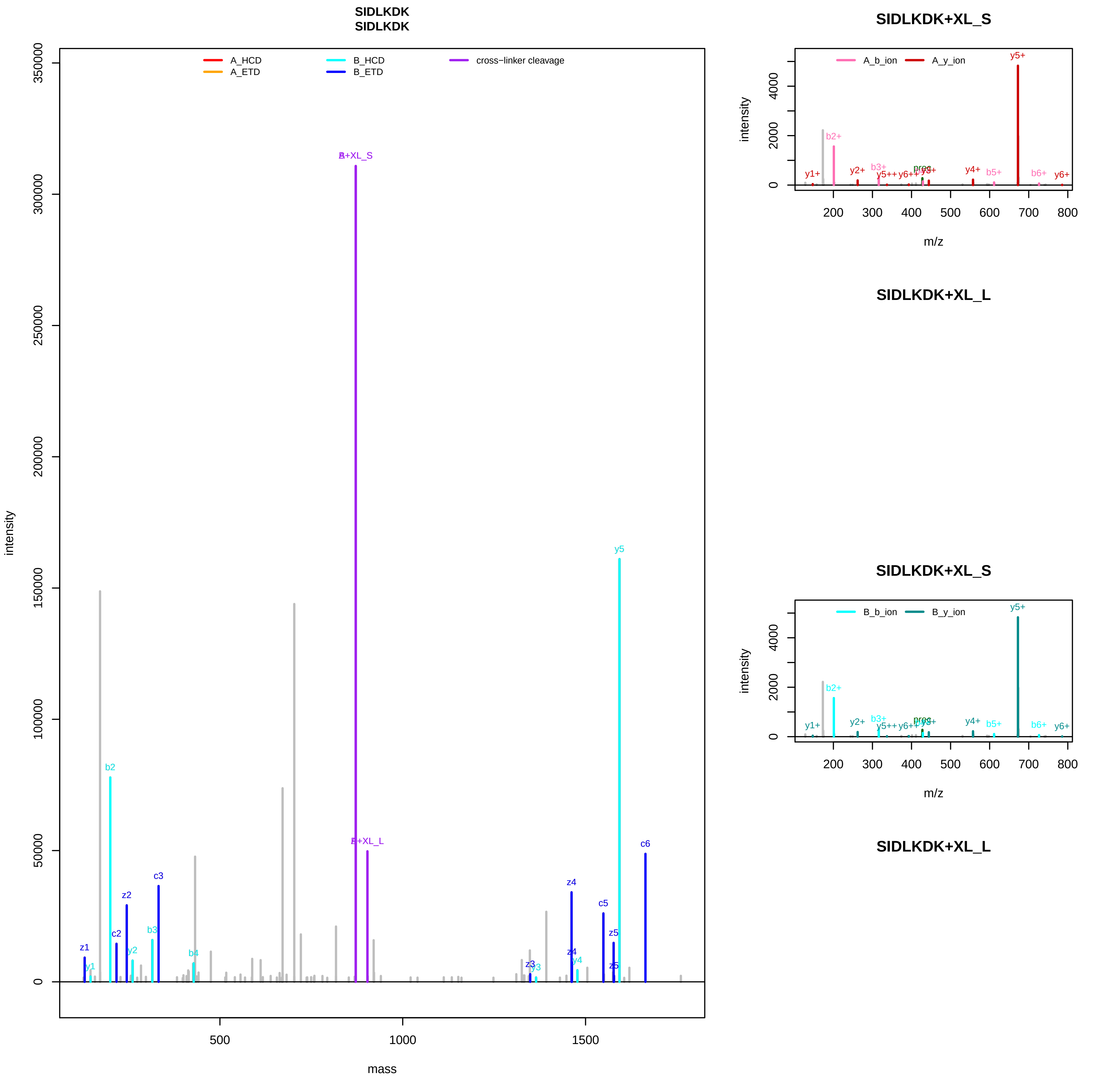
SIDLKDK
SIDLKDK
A_HCD
A_ETD
B_HCD
B_ETD
cross−linker cleavage
intensity
mass
B+XL_S
B+XL_L
y5
b2
c6
z4
c5
z5
c3
z2
c2
z1
b3
y2
y1
b4
z3
y3
y4
SIDLKDK+XL_S
A_b_ion
A_y_ion
y5+
b2+
y1+
y2+
b3+
y5++
y6++
prec
y3+
y4+
b5+
b6+
y6+
m/z
SIDLKDK+XL_L
SIDLKDK+XL_S
B_b_ion
B_y_ion
SIDLKDK+XL_L
